# Study on hand disinfection in inpatient geriatric care on the superiority of cold plasma aerosol versus alcohol-based disinfection methods in a parallel group design

**DOI:** 10.1038/s41598-024-72524-7

**Published:** 2024-09-17

**Authors:** Tim Tischendorf, Tom Schaal, Ulrich Schmelz

**Affiliations:** 1 Faculty of Health and Healthcare Sciences, University of Applied Sciences Zwickau, Zwickau, Germany; 2 University of Fulda, Fulda, Germany

**Keywords:** Cold plasma, Hygiene conformity, Safety, Skin protection, Disinfection, Plasma physics, Health care

## Abstract

The introduction of fundamental hygiene protocols within the healthcare sector during the nineteenth century led to a significant reduction in mortality rates. Contemporary advancements, such as alcohol-based sanitizers, have further enhanced hand hygiene practices. However, these measures are often overlooked in nursing facilities, resulting in low staff compliance rates and increased cross-infection rates. Novel approaches, such as cold plasma hand disinfection, present promising alternatives due to their minimal skin damage and economic benefits. This study aims to compare the disinfectant efficacy of cold plasma aerosol under practical application conditions with an alcoholic hand disinfectant listed by the Association for Applied Hygiene. The microbial count on participants’ hands was measured, with particular attention paid to the spontaneous occurrence of fecal indicators and the presence of potentially infectious bacteria. A t-test for independent samples was conducted to determine whether there was a significant difference between the two cohorts regarding the research question. Statistical analysis revealed that the mean log colony-forming unit (CFU) values were significantly lower in the test cohort using only the cold plasma method for hand disinfection compared to the cohort using conventional alcohol-based hand disinfection. Moreover, it was demonstrated that, unlike alcohol-based hand disinfection, cold plasma application ensures the effective elimination of *Staphylococcus aureus*. The findings indicate that staff utilizing plasma disinfection have an average bacterial count that is 0.65 log units lower than those who regularly use alcohol-based hand disinfection. In addition to the efficacy of cold plasma disinfection, its superiority over alcohol-based hand disinfection was also established. Beyond offering economic and logistical advantages, cold plasma disinfection provides additional health benefits as it does not induce skin damage, unlike alcohol-based hand disinfection.

## Introduction

In the nineteenth century, the healthcare sector witnessed a revolution in hygiene practices, marked by the introduction of basic cleanliness, disinfecting handwashing, and sterilization of surgical instruments, which brought about a significant reduction in mortality rates. These methods are in line with the germ theory of infection causation^1,2^. Further advancements introduced chemical and thermal sterilization, proper cleaning of textiles, and the utilization of disposable gloves. Leading institutes in the United States and Germany acknowledge hand hygiene as highly effective in preventing nosocomial infections^3^. The advent of alcohol-based disinfectants enhanced compliance with hand hygiene, thereby reducing healthcare-associated infections and antibiotic-resistant pathogens.

Hand hygiene is a fundamental measure for infection prevention, eliminating transient germs acquired through contact with patients and surfaces. Alcohol serves as the active ingredient, with concentrations of 60–80% alcohol sufficing for routine use, while 99% alcohol is necessary for specific viruses such as Noroviruses. However, acceptance of hand disinfection in nursing homes remains low, often attributed to factors such as convenience, lack of training, time constraints, skin problems, and lack of awareness^4,5^.

Research by Smith et al. and Cordeiro et al.^6,7^ revealed that hand hygiene compliance among staff in long-term care facilities averages around 14%, with variations observed before and after patient contact. Using real data on Norovirus transmission, Assab and Temime demonstrated that raising this compliance rate to 60% could result in a 75% reduction in gastrointestinal diseases among residents within 100 days^8^. A Cochrane Review by Gould et al. reported mixed results concerning the effectiveness of interventions designed to improve hand hygiene, with most increases being modest yet significant. Despite the inability to isolate the most effective intervention, all measures appear to contribute to some degree in enhancing hand hygiene compliance, with the potential drawback of resource misallocation^9^.

A more nuanced perspective highlights unique challenges in infection control within long-term care facilities, with hygiene officers encountering difficulties in applying various recommendations (e.g. DGKH (German Society for Hospital Hygiene (Deutsche Gesellschaft für Krankenhaushygiene e.V.)), KRINKO (Commission for Hospital Hygiene and Infection Prevention at the Robert Koch Institute), DQNP (German Network for Quality Development in Nursing)) to their respective work areas. There is an urgent need for evidence-based hygiene guidelines tailored to these specific facilities and their legal relationships with residents. Standardization and monitoring by authorities and institutions such as KRINKO are also warranted. The revised Infection Protection Act (IfSG) now entrusts health authorities with oversight of outpatient nursing services and home-like service providers^10^.

Emerging studies reveal the potential of cold plasma hand disinfection in clinical and healthcare settings, positioning it as an alternative to conventional alcohol-based hand disinfection^11–13^. The use of cold plasma not only minimizes skin exposure but also preserves skin integrity even after repeated applications, contributing to improved skin health^13^. Findings suggest that incorporating cold plasma disinfection could become a cornerstone in hospital and nursing home hygiene, particularly in care and treatment areas^11,12^. Moreover, the technology presents substantial economic benefits and the potential for antimicrobial treatment of contaminated wounds and skin^12,13^.

**Fig. 1:**
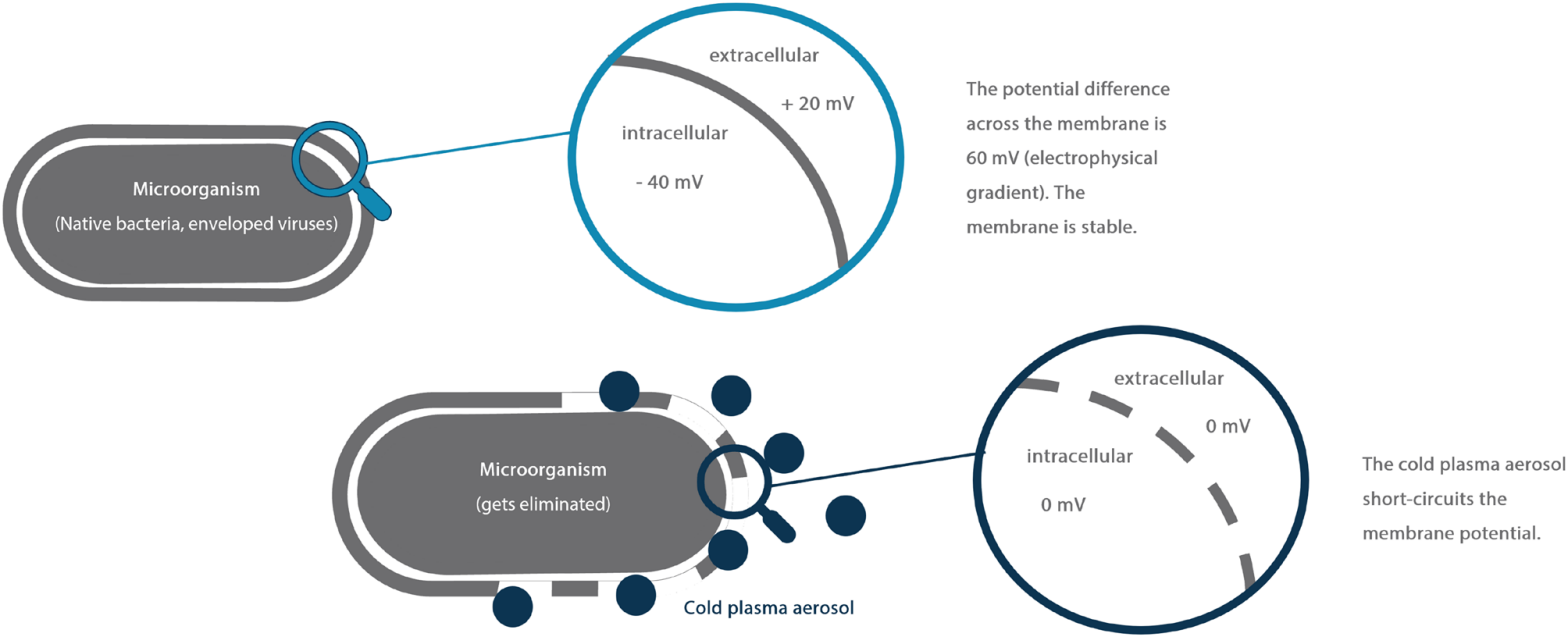
Cold plasma eliminates microorganisms by a short circuit of the membrane potential (own illustration based on^15^).

In cold plasma aerosol reactions (atmospheric low-temperature plasma), hydroxyl radicals are formed, which can alter the electrophysiological potential of microorganisms’ plasma membranes, disrupting transport processes and causing lethal damage^14,15^. Secondary oxidation effects can also induce plasma membrane leakage, further compromising microorganism function^13^. These electrophysical effects underpin cold plasma’s antimicrobial properties (see Fig. 1). The aerosol’s liquid phase, enriched with plasma reaction products, ensures maximum dispersion in the gaseous phase^14^.

Notably, cold plasma poses no acute or chronic toxicity to eukaryotic tissues (plants, animals, and humans). This is attributed to the fact that antioxidant enzymes in these tissues neutralize plasma reaction products before they can cause harm to eukaryotic cells^12,13^. The medical safety of cold plasma is supported by toxicity assessments, such as the Ames test (EN ISO 10993-3) and a Cytotoxicity test (EN ISO 10993-5), which the examined cold plasma process (in the form of PLASMOHAND) passed, showing no mutagenic effects in human cells. Furthermore, the ozone emissions from the examined device comply with regulatory standards, with a time-weighted average of 39 µg per cubic meter over eight hours, well below the 120 µg per cubic meter limit, thereby confirming its environmental compatibility^16^.

Atmospheric low-temperature plasma generates hydroxyl radicals from ambient air, which contains all essential reactants (oxygen and water vapor). The plasma reaction is an electrophysical process in which an ignition pulse renders the gas mixture conductive. A subsequent low current flow at a defined voltage and frequency induces the desired reaction of the atmospheric low-temperature plasma, producing effective hydroxyl radicals as reaction byproducts^17,18^. This discovery has led to the development of a device that harnesses these principles for hand disinfection, encompassing the entire process within a single system^18^.

The aerosol application in the design of the device eliminates the problematic wetting gaps associated with alcoholic hand disinfectants, enabling more complete and homogeneous hand exposure. Residual moisture on hands does not interfere with the process, as studies indicate no adverse effects on plasma disinfection efficacy.

The current investigation aims to comparatively assess the disinfectant efficacy of the cold plasma aerosol under practical application conditions, in comparison to an alcoholic hand disinfectant listed in the VAH (Association for Applied Hygiene) list. The difference in efficacy between alcohol-based hand disinfection and cold plasma-based hand disinfection will be measured in terms of reduction in log colony-forming units. Additionally, the spontaneous occurrence of fecal indicators (*Enterococcus spp.*) and the presence of potentially infectiologically problematic bacteria (*Staphylococcus aureus*) on the hands will be examined.

## Methods

### Principle and methodology of the plasma disinfection process to be tested

The device under investigation generates an aerosol within the device by adding liquid particles (distilled water) using an ultrasonic nebulization process. The aerosol is then fed into a mixing chamber where it is conditioned by plasma reaction products generated from ambient air via a corresponding plasma source. The plasma reaction products increase the electrophysical potential of the nebulized liquid without any material change. The aerosol is subsequently fed into a chamber for eight seconds, during which the microorganisms on the user’s hands are eliminated. The device is configured as a box into which hands are introduced under a “no-touch” principle, automatically activating the device. The user’s hands, with spread fingers, are exposed to the aerosol containing plasma reaction byproducts for an eight-second duration, after which the disinfection process is complete.

### Sampling

Sampling is conducted identically for both cohorts. Fifteen nurses from each facility are spontaneously recruited during their daily activities after four to five hours of work in the late morning. The experiment was conducted in one day, and the facility staff were unaware of whether or when such an experiment would be conducted. Participants in cohort A regularly used an approved alcohol-based preparation (CimoCid Plus, Dr. Schnell GmbH & Co.KG a. A, Munich) according to the VAH list in accordance with the hygiene plan of the care facility, while participants in cohort B carried out hand disinfection with the plasma disinfection device under investigation instead of the alcohol-based preparation. If the analysis indicates that the microbial densities of both test groups are essentially the same, or if cohort B shows an even lower microbial count than cohort A, it would demonstrate that the effectiveness of the plasma disinfection method is equivalent to that of the alcohol disinfection method. In this context, equivalence refers to the metrological traceability to a reference standard, which is the alcohol-based disinfectant listed by the VAH as per the facility’s hygiene plan. Cohorts are recruited spontaneously, ideally without any intentional hand disinfection immediately before testing, allowing for the determination of the average microbial count on hands under typical working conditions in the facility^19,20^.

The sampling includes 15 hand samples from each of the two cohorts. For this purpose, 15 Petri dishes with a diameter of 90 mm are provided for each of the two cohorts, each containing 10 ml of sterile 0.9% NaCl solution. The EN 1500 standard method is not fully applicable to the plasma disinfection method since the norm pertains to liquid, chemically based hand disinfectants, whereas the currently examined method is effective through the gas phase. The *E. coli* test contamination provided for in EN 1500 was deliberately omitted; instead, the bacterial load of the hands was compared with the alcohol preparation during the ongoing application of the plasma disinfection procedure.

Subsequently, the fingertips of the long fingers of the dominant hand of the sampled person are pressed against the thumb in the liquid in the Petri dish (approximately 30 s), suspending the resident and transient flora into a liquid for further examination^21^. The Petri dishes are then sealed and arbitrarily numbered from 01 to 15, or from 16 to 30, ensuring a blinded and anonymized examination. Immediately after suspending the fingers in the Petri dishes, the dilution series was prepared, with a maximum time interval of five minutes. The experimental protocols were approved by Dr Schmelz GmbH Malsfeld and Umwelthygiene Marburg GmbH & Co. KG. The laboratory work and the laboratory organization are accredited according to DIN EN ISO 17025 for microbiological tests on medical devices and medicinal products.

The test subjects have declared that their participation in the test is voluntary, with no direct dependencies, and that they are not, do not want to be, or cannot be pregnant. All methods of this study were conducted in accordance with the relevant guidelines and regulations. The participating nurses provided their informed consent to participate in the study in advance.

### Cultivation of aerobic, mesophilic total microbial count

From each dilution step, 100 μl is applied to a Petri dish containing Caso Agar and evenly distributed with a Drigalski spatula. The plates are aerobically cultivated at 36 °C for 48 h in an incubator^22^.

### Cultivation of *Enterococcus* spp. strains as fecal indicators

Given the low expectation of the occurrence of fecal indicators with regular professional implementation of hygienic hand disinfection, a volume of 100 μl of the 10^–2^ dilution is applied to a Slanetz-Bartley agar by direct inoculation in the Petri dish and uniformly distributed with the Drigalski spatula. The Slanetz-Bartley Agar is aerobically incubated at 44 °C for 48 h, followed by further cultivation for up to 72 h^23^.

### Cultivation of *S. aureus* strains

Qualitative detection is achieved by transferring 5 ml of the initial hand wash suspension into Petri dishes containing Mannitol Salt Agar in the form of a nutrient card disc. The cultivation is conducted aerobically at 36 °C for 48 h. Yellow discoloration indicates the presence of *S. aureus*. Subsequently, a subculture is established, and the grown colonies are examined for catalase formation and clumping factor to confirm the findings^24^.

### Statistical analysis

The collected data were tested for normal distribution using the Shapiro–Wilk test. The independent samples t-test was calculated to answer the research hypothesis (t-test, *p* < 0.05, two-tailed). The Levene test was used to test for homogeneity of variance. All statistical analyses were performed using IBM SPSS Statistics version 29.0.0.0 (MacOS)^25^.

## Results

Two hand disinfection methods, namely alcohol-based hand disinfection and cold plasma hand disinfection, were analyzed in terms of the average number of log colony-forming units. Additionally, the spontaneous occurrence of fecal indicators (*Enterococcus spp.*) and the presence of potentially infectious bacteria (*S. aureus*) on the hands were investigated.

The collected data showed normal distribution (*p* = 0.113; Shapiro–Wilk Test). The Levene test conducted before the t-test revealed a homogeneity of variance (*p* = 0.214). The t-test for two independent samples showed that cohort B (M = 3.42) had significantly lower mean values of log colony-forming units than cohort A (M = 4.07) (*p* = 0.045, two-sided). This indicates that there is a difference in the mean values of log colony-forming units between the two disinfection methods, favoring the plasma disinfection method.

**Fig. 2:**
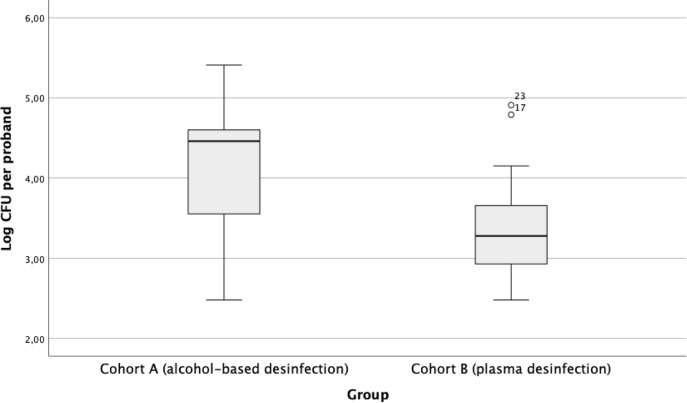
Comparison of the bacterial count between cohort A and B. The plasma disinfection method shows lower median log CFU values than the alcohol-based disinfection method. The interquartile range is larger in cohort A, while cohort B is less variable and has two outliers (B17, B23) above the upper whisker, indicating unusually high log CFU values for these subjects. *Note. CFU* = *Colony Forming Units.*

The following figures illustrate the comparison of the plasma disinfection method (cohort B; n = 15) for hygienic hand disinfection with the alcohol-based disinfection method (cohort A; n = 15). The boxplot diagram (Fig. 2) illustrates the distribution of log colony-forming units (log CFU) per test person for the two cohorts.

**Fig. 3:**
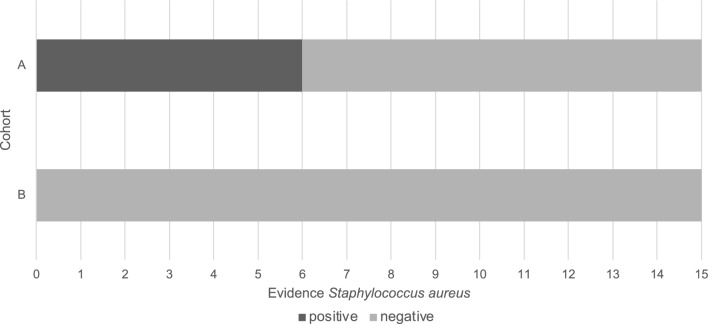
Comparison of the evidence of *S. aureus* per cohort. *Note. Cohort A* = *alcohol-based disinfection. Cohort B* = *plasma disinfection.*

From a microbiological and medical specialist perspective, the lower bacterial count observed on the hands of cohort B, compared to cohort A, demonstrates the effectiveness of the plasma disinfection procedure in terms of disinfection. Furthermore, no *S. aureus* was detected in cohort B, in contrast to cohort A (Fig. 3). This indicates that cold plasma application ensures the effective elimination of *S. aureus*, which is not the case with alcohol-based hand disinfection.

A clear and transparent tabular presentation of the data is included in the appendix.

## Discussion

Test cohort A represents the average microbial count on hands when using the established alcohol-based hand disinfectant, as per the facility’s hygiene plan and the VAH list. Under regular hand disinfection with alcohol, an average microbial count of log 4.07, which is equivalent to 104.07 or 12,000 CFU, is observed on the examined areas of the hands. *Enterococcus spp.* was not detected in any participants. *S. aureus* was qualitatively identified in the resident flora in six out of 15 participants.

Test cohort B demonstrates the average microbial count on hands when using the investigated plasma disinfection method. An average microbial count of log 3.42, which is equivalent to 103.42 or 2600 CFU, is observed on the examined areas of the hands. *Enterococcus spp.* was not detected in any participants, and *S. aureus* was also not detected in any participants.

The results indicate that personnel utilizing plasma disinfection exhibit an average microbial count that is 0.65 log units lower than the microbial count on hands when regularly applying alcohol-based hand disinfection, according to the VAH list and the facility’s hygiene plan. The two independent samples t-test was used to demonstrate that this difference in the average number of germs was also significant. This proves that the plasma disinfection method investigated is not only equivalent in practical application to alcohol-based hand disinfection in terms of the research hypothesis, but even superior. Accordingly, the plasma disinfection method leads to a 0.65 log unit lower mean bacterial count on the hands than the use of alcohol-based hand sanitizers^13^. Therefore, from the perspective of hygiene and microbiology, it is feasible to consider the alternative hand disinfection method based on plasma disinfection as equivalent to alcohol-based hand disinfection^26^. As Test cohort A employs a chemically approved hand disinfectant listed by the VAH, according to the department’s hygiene plan, this test establishes metrological traceability of Test cohort B to Test cohort A, allowing the plasma disinfection method to be considered at least equivalent to alcohol-based hand disinfection.

In the overall view of the results, Test cohort B exhibits no *S. aureus* colonization or contamination on the hands of participants. This suggests the effectiveness of the disinfection method, validating the quantitative results of the aerobic mesophilic total microbial count when comparing cohorts A and B^27^.

Regarding the discussion of methods, the sample size of 15 subjects per cohort and the fact that the trial was only carried out on one day must be taken into account. Future studies could therefore not only increase the sample size, but also extend the trial to several days or weeks. A research desideratum would therefore be the extent to which the results of this study can be further quantified with larger sample sizes and over a longer study period. Regarding the culture technique, this is a widely used standardized methodological procedure that ensures the reproducibility of the results.

## Conclusion

The plasma disinfection method under investigation has been shown to be superior to the alcohol-based disinfection method as per the VAH list. The effectiveness of the plasma disinfection method for microbiological disinfection has been securely established.

The use of the tested cold plasma disinfection process offers several advantages in addition to its proven effectiveness^28^. Firstly, the effectiveness is independent of hand moisture, meaning that even hands that have been washed immediately beforehand can be reliably disinfected. Secondly, no skin-irritating or skin-damaging effects have been identified^21^. Furthermore, the process ensures even wetting of the hands without the gaps that can occur with alcohol-based or other liquid sanitizers^29,30^.

Another advantage is that the active ingredient is produced directly in the device, and no additional chemicals are required^31^. With the refillable water tank, approximately 2000 disinfection cycles can be carried out, which theoretically corresponds to more than 15 plastic bottles with 500 ml of hand sanitizer each. This simplifies the internal logistics of the nursing home and reduces the environmental footprint, representing small innovative steps towards a green nursing home^32^.

In addition, the cold plasma disinfection process enables a time saving of eight seconds compared to 30 s for alcohol-based hand disinfection. Together with the proven effectiveness, these procedural advantages are features that make the process a reliable and safe alternative to alcohol-based hand disinfection.

## Supplementary Information

Supplementary Table.

## Data Availability

All data generated or analysed during this study are provided within the manuscript itself.
