# Supplementary material for: Study on hand disinfection in inpatient geriatric care on the superiority of cold plasma aerosol versus alcohol-based disinfection methods in a parallel group design
Source: Sci Rep. 2024 Sep 17;14:21703. doi: 10.1038/s41598-024-72524-7 (PMC11408535; doi:10.1038/s41598-024-72524-7)
Supplement: Supplementary file 1 — Supplementary Table. [file 41598_2024_72524_MOESM1_ESM.docx]

Appendix. Comparison of the plasma disinfection process for hygienic hand disinfection with alcohol-based disinfection.

|  | **Aerobic mesophilic total bacterial count** | | | | | | | | | ***Enterococcus spp.* as faecal indicator** | | | | | | ***S. aureus*** |
| --- | --- | --- | --- | --- | --- | --- | --- | --- | --- | --- | --- | --- | --- | --- | --- | --- |
| **No.:** | **V1** | **V2** | **CFU/ plate** | **CFU/ approach** | **Dilution** | **CFU/ Proband** | **Log CFU per Proband** | **mean Log CFU/Proband** | **Log difference from alcohol to plasma** | **V1** | **V2** | **CFU/ plate** | **CFU/ approach** | **Dilution** | **CFU/ Proband** | **Evidence pos. / neg.** |
| A01 | 10,0 ml | 0,1 ml | 16 | 1600 | 10 | 16000 | **4,20** |  |  | 10,0 ml | 0,1 ml | 0 | 0 | 10 | 0 | positive |
| A02 | 10,0 ml | 0,1 ml | 48 | 4800 | 1 | 4800 | **3,68** |  |  | 10,0 ml | 0,1 ml | 0 | 0 | 10 | 0 | positive |
| A03 | 10,0 ml | 0,1 ml | 31 | 3100 | 10 | 31000 | **4,49** |  |  | 10,0 ml | 0,1 ml | 0 | 0 | 10 | 0 | negative |
| A04 | 10,0 ml | 0,1 ml | 72 | 7200 | 10 | 72000 | **4,86** |  |  | 10,0 ml | 0,1 ml | 0 | 0 | 10 | 0 | negative |
| A05 | 10,0 ml | 0,1 ml | 26 | 2600 | 100 | 260000 | **5,41** |  |  | 10,0 ml | 0,1 ml | 0 | 0 | 10 | 0 | negative |
| A06 | 10,0 ml | 0,1 ml | 4 | 400 | 1 | 400 | **2,60** |  |  | 10,0 ml | 0,1 ml | 0 | 0 | 10 | 0 | positive |
| A07 | 10,0 ml | 0,1 ml | 32 | 3200 | 10 | 32000 | **4,51** |  |  | 10,0 ml | 0,1 ml | 0 | 0 | 10 | 0 | positive |
| A08 | 10,0 ml | 0,1 ml | 68 | 6800 | 1 | 6800 | **3,83** |  |  | 10,0 ml | 0,1 ml | 0 | 0 | 10 | 0 | negative |
| A09 | 10,0 ml | 0,1 ml | 38 | 3800 | 10 | 38000 | **4,58** |  |  | 10,0 ml | 0,1 ml | 0 | 0 | 10 | 0 | negative |
| A10 | 10,0 ml | 0,1 ml | 42 | 4200 | 10 | 42000 | **4,62** |  |  | 10,0 ml | 0,1 ml | 0 | 0 | 10 | 0 | positive |
| A11 | 10,0 ml | 0,1 ml | 29 | 2900 | 10 | 29000 | **4,46** |  |  | 10,0 ml | 0,1 ml | 0 | 0 | 10 | 0 | negative |
| A12 | 10,0 ml | 0,1 ml | 21 | 2100 | 100 | 210000 | **5,32** |  |  | 10,0 ml | 0,1 ml | 0 | 0 | 10 | 0 | negative |
| A13 | 10,0 ml | 0,1 ml | 3 | 300 | 1 | 300 | **2,48** |  |  | 10,0 ml | 0,1 ml | 0 | 0 | 10 | 0 | negative |
| A14 | 10,0 ml | 0,1 ml | 4 | 400 | 1 | 400 | **2,60** |  |  | 10,0 ml | 0,1 ml | 0 | 0 | 10 | 0 | negative |
| A15 | 10,0 ml | 0,1 ml | 27 | 2700 | 1 | 2700 | **3,43** | **4,07** |  | 10,0 ml | 0,1 ml | 0 | 0 | 10 | 0 | positive |
| B16 | 10,0 ml | 0,1 ml | 39 | 3900 | 1 | 3900 | **3,59** |  |  | 10,0 ml | 0,1 ml | 0 | 0 | 10 | 0 | negative |
| B17 | 10,0 ml | 0,1 ml | 61 | 6100 | 10 | 61000 | **4,79** |  |  | 10,0 ml | 0,1 ml | 0 | 0 | 10 | 0 | negative |
| B18 | 10,0 ml | 0,1 ml | 14 | 1400 | 10 | 14000 | **4,15** |  |  | 10,0 ml | 0,1 ml | 0 | 0 | 10 | 0 | negative |
| B19 | 10,0 ml | 0,1 ml | 12 | 1200 | 1 | 1200 | **3,08** |  |  | 10,0 ml | 0,1 ml | 0 | 0 | 10 | 0 | negative |
| B20 | 10,0 ml | 0,1 ml | 5 | 500 | 1 | 500 | **2,70** |  |  | 10,0 ml | 0,1 ml | 0 | 0 | 10 | 0 | negative |
| B21 | 10,0 ml | 0,1 ml | 15 | 1500 | 1 | 1500 | **3,18** |  |  | 10,0 ml | 0,1 ml | 0 | 0 | 10 | 0 | negative |
| B22 | 10,0 ml | 0,1 ml | 52 | 5200 | 1 | 5200 | **3,72** |  |  | 10,0 ml | 0,1 ml | 0 | 0 | 10 | 0 | negative |
| B23 | 10,0 ml | 0,1 ml | 82 | 8200 | 10 | 82000 | **4,91** |  |  | 10,0 ml | 0,1 ml | 0 | 0 | 10 | 0 | negative |
| B24 | 10,0 ml | 0,1 ml | 32 | 3200 | 1 | 3200 | **3,51** |  |  | 10,0 ml | 0,1 ml | 0 | 0 | 10 | 0 | negative |
| B25 | 10,0 ml | 0,1 ml | 16 | 1600 | 1 | 1600 | **3,20** |  |  | 10,0 ml | 0,1 ml | 0 | 0 | 10 | 0 | negative |
| B26 | 10,0 ml | 0,1 ml | 24 | 2400 | 1 | 2400 | **3,38** |  |  | 10,0 ml | 0,1 ml | 0 | 0 | 10 | 0 | negative |
| B27 | 10,0 ml | 0,1 ml | 4 | 400 | 1 | 400 | **2,60** |  |  | 10,0 ml | 0,1 ml | 0 | 0 | 10 | 0 | negative |
| B28 | 10,0 ml | 0,1 ml | 19 | 1900 | 1 | 1900 | **3,28** |  |  | 10,0 ml | 0,1 ml | 0 | 0 | 10 | 0 | negative |
| B29 | 10,0 ml | 0,1 ml | 3 | 300 | 1 | 300 | **2,48** |  |  | 10,0 ml | 0,1 ml | 0 | 0 | 10 | 0 | negative |
| B30 | 10,0 ml | 0,1 ml | 6 | 600 | 1 | 600 | **2,78** | **3,42** |  | 10,0 ml | 0,1 ml | 0 | 0 | 10 | 0 | negative |
|  |  |  |  |  |  |  |  |  | **0,65** |  |  |  |  |  |  |  |
| Note. No.= Subject number. CFU=Colony Forming Units. Spp.=species. S.=Staphylococcus. Pos.=positive. Neg=negative. | | | | | | | | | | | | | | | | |
